# NeuriteQuant: An open source toolkit for high content screens of neuronal Morphogenesis

**DOI:** 10.1186/1471-2202-12-100

**Published:** 2011-10-11

**Authors:** Leif Dehmelt, Gunnar Poplawski, Eric Hwang, Shelley Halpain

**Affiliations:** 1 Max-Planck-Institute of Molecular Physiology, Otto-Hahn-Str. 11, 44227 Dortmund, Germany and Dortmund University of Technology, Otto-Hahn-Str. 6, 44227, Dortmund, Germany; 2 Division of Biological Sciences, University of California, San Diego, La Jolla, CA 92093, USA; 3 Department of Biological Science and Technology, National Chiao Tung University, Hsinchu, Taiwan 30068

## Abstract

**Background:**

To date, some of the most useful and physiologically relevant neuronal cell culture systems, such as high density co-cultures of astrocytes and primary hippocampal neurons, or differentiated stem cell-derived cultures, are characterized by high cell density and partially overlapping cellular structures. Efficient analytical strategies are required to enable rapid, reliable, quantitative analysis of neuronal morphology in these valuable model systems.

**Results:**

Here we present the development and validation of a novel bioinformatics pipeline called NeuriteQuant. This tool enables fully automated morphological analysis of large-scale image data from neuronal cultures or brain sections that display a high degree of complexity and overlap of neuronal outgrowths. It also provides an efficient web-based tool to review and evaluate the analysis process. In addition to its built-in functionality, NeuriteQuant can be readily extended based on the rich toolset offered by ImageJ and its associated community of developers. As proof of concept we performed automated screens for modulators of neuronal development in cultures of primary neurons and neuronally differentiated P19 stem cells, which demonstrated specific dose-dependent effects on neuronal morphology.

**Conclusions:**

NeuriteQuant is a freely available open-source tool for the automated analysis and effective review of large-scale high-content screens. It is especially well suited to quantify the effect of experimental manipulations on physiologically relevant neuronal cultures or brain sections that display a high degree of complexity and overlap among neurites or other cellular structures.

## Background

High content screening (HCS) of cells based on morphological parameters is increasingly used to identify novel molecular pathways in disease or potential new therapeutic treatments. Screens targeting neuronal development or neurodegeneration in particular aim to quantify neurites (axons and dendrites). Manual analysis of neuronal morphology is time consuming and becomes impractical for large datasets. While specialized commercial software applications are available to measure neurite outgrowth, such tools are usually not openly available for user customization beyond the supplied standard interface. On the other hand, free software tools for quantitative analysis of neuronal morphology do not offer convenient automated analysis of large-scale data sets (such as those produced by genome-wide RNA interference-based screens or extensive compound library screens), and often require a significant level of user interaction [1,2].

**Figure 1:**
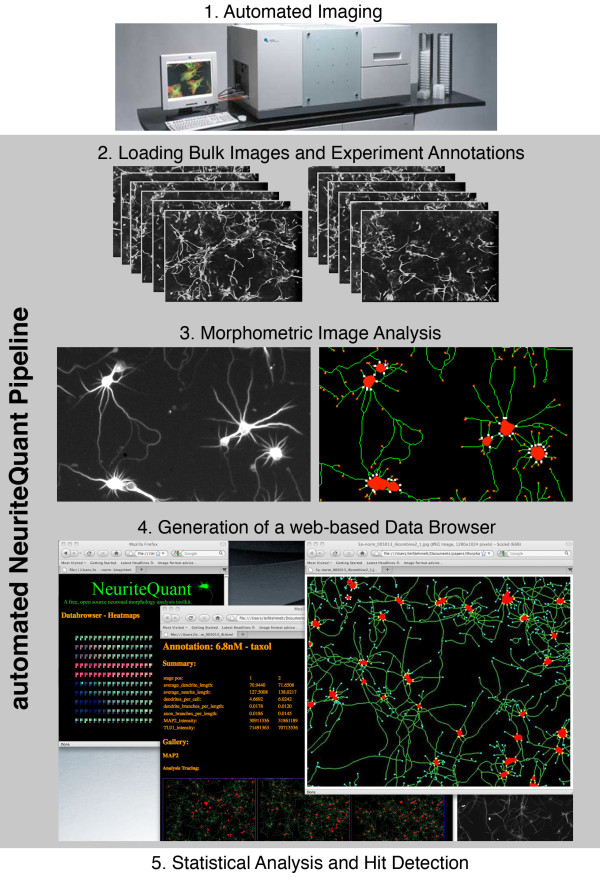
**Flow Diagram of the NeuriteQuant Content Pipeline**.

Here, we describe and make freely available a bioinformatics toolkit we term "NeuriteQuant" to perform automated analysis of neurite outgrowth and branching. The toolkit is open-source and based on the free image analysis software program ImageJ. Unlike other non-commercial approaches for neuronal analysis, the NeuriteQuant pipeline provides a complete, integrated routine to facilitate genome-wide high-content analysis as well as small-scale experiments. NeuriteQuant is easily configured to process large, complex datasets produced by automated screening microscopes (Figure 1). Results are automatically organized into a web-based data browser, which provides detailed graphical representations of neuronal morphological measurements, as well as links to the raw images. NeuriteQuant does not require any additional commercial software products, and is easily adapted to interface with ImageJ compatible data files produced by automated microscopy systems. The open-source concept of NeuriteQuant facilitates rapid development of related cell-based morphological analyses, which will be made freely available on the NeuriteQuant website [3].

The toolkit can be applied to measure neuronal differentiation, neurite outgrowth, branching and the polarization of neurites into axons and dendrites. It is optimized for images containing dozens of neurons per field of view and multiple fields per condition, so that each experimental data point represents hundreds of neurons. A key feature of NeuriteQuant is that it quantifies neurite and cell body information based on morphological criteria, not on signal intensity. The algorithm applies a series of morphological filters, culminating in quantitative measurements of neurite length, neuronal cell body area, neurite-cell body attachment points, and neurite endpoints per field. From these primary measurements, average measurements per neuron are derived for neurite length, cell body area, branch points, and neurite count.

## Implementation

The majority of the NeuriteQuant tool is implemented as an ImageJ macro, and can be easily manipulated using a simple text editor. Reference for the ImageJ macro language is available online [4]. Additional functionality that could not be implemented as an ImageJ macro was added in the form of ImageJ plugins using the programming language Java. The source code for these custom-made plugins is also included in the NeuriteQuant package.

### Overview of the NeuriteQuant Analysis Pipeline

A particular strength of NeuriteQuant is its flexibility due to open source implementation. This flexibility allows easy adaptation of different data sources from various imaging platforms. In addition, the toolkit is already configured with a powerful content pipeline, which facilitates streamlined management of image and annotation data (Figure 1).

In general, image data must be provided according to a naming convention that identifies individual experimental conditions (for example by well position, plate identifier and/or other classifiers). For this study we controlled a standard inverted light microscope equipped with automated filter-cube turret and automated x-y-z stage positioning using custom Metamorph journals (available upon request) to automatically generate sets of images.

It is advantageous, especially for large-scale experiments, to evaluate and review automated analysis data rapidly and efficiently. NeuriteQuant fills this need by automatically creating an autonomous, web-based data browser for each analysis run (see [5] for an example) that facilitates review by a human observer. This data browser serves as a platform for visualization and sharing of experimental results. It provides easy access to compressed versions of the original image data, incorporates user-defined experiment annotations, tracing of morphological image features, a customizable, interactive three-colour graphical representation of quantitative analysis in the form of so-called heatmaps, and interactive 2-D graphical plots. Finally, NeuriteQuant exports all measurement data into tab-delimited text files, which can be easily imported into statistics packages for subsequent analysis and hit detection (see [6] for detailed instructions).

### Neuromorphometric Measurement Algorithm

**Figure 2:**
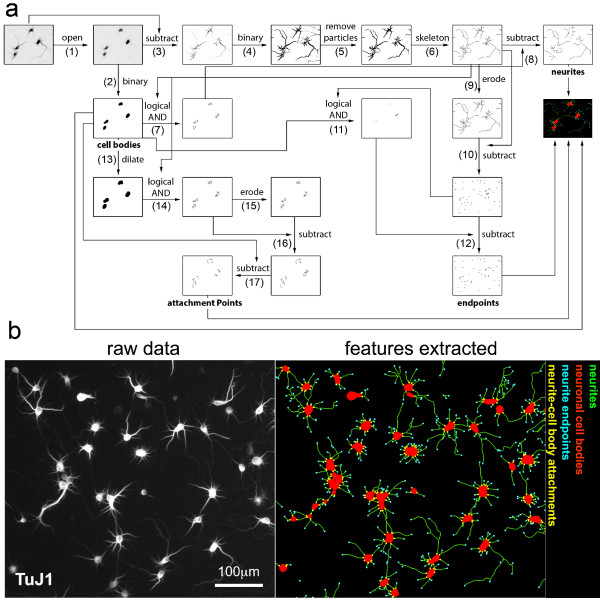
**Image Processing Algorithm for Quantification of Neuronal Morphology**. a) Flow diagram that illustrates the extraction of key morphological features from single colour images of neurons stained with antibodies to either the neuron-specific marker betaIII-tubulin or the dendrite-specific marker MAP2. Numbers refer to steps in the algorithm description (Main Text: Implementation). A summary of the analysis algorithm is provided in the Additional File 1. b) Example of feature extraction from cultured primary hippocampal neurons. The majority of neuronal structures are identified accurately by this procedure. The analysis algorithm quantifies total neurite length by counting pixels of the skeletonized neurites (green) and total neuronal cell body area per field of view (red). In addition, the algorithm identifies and counts the majority of cell bodies, neurite endpoints (cyan) and neurite-cell body attachment points (yellow), as well as the total staining intensities per field. These values are used to deduce additional measurements (see text for details).

Our method for morphological analysis is highly sensitive and largely independent of signal intensities, and thus detects both neurites that contain abundant signal as well as those that are barely detectable above background. This minimal signal dependence for neurite detection is achieved by using the public domain Greyscale Morphology filter by Dimiter Prodanov (Université catholique de Louvain, Brussels), which can be used to selectively enhance either small, neurite-like structures or globular, cell body-like structures in the image (Figure 2). First, circular objects, which usually represent cell bodies, are amplified using an open filter (step 1) and isolated by binarization (step 2). Fiber-like structures, such as neurites, are identified by subtraction (step 3) of the open filtered image from the original image and subsequent binarization (step 4). Due to the strong enhancement of neurite or cell body structures, variations of staining intensities minimally affect the detection procedure and therefore a single, preset threshold can be used for binarization of all images of an individual set of experiments. This threshold can either be obtained interactively via a guided procedure provided in NeuriteQuant or set manually for a given set of images.

Small structures, which usually represent debris or imaging artefacts, are excluded by rapid size filtering (step 5), which was implemented by an altered flood-fill algorithm originally included in the ImageJ package. In contrast to the standard particle analyzer built in ImageJ, this modified filter is able to filter objects enclosed by larger objects (for example small objects enclosed by neurite loops). Subsequently, a well defined, one pixel wide representation of the fibrous structure is obtained by the skeletonization function of ImageJ (step 6). Skeletonized fibrous structures that do not belong to neurites are often also found within neuronal cell bodies - therefore, the overlap between fibre structures and neuronal cell bodies is determined (step 7) and subtracted (step 8) to yield a clean, one pixel-wide representation of neurites. Due to this filter, neurites that grow on top of neuronal cell bodies are also excluded from our analysis.

To identify the number of neurite endpoints, the single terminal pixel of the skeletonized fiber structures are eroded (step 9) and subtracted from the original skeleton (step 10). By subsequent subtraction (step 12) of the skeleton/cell body overlap (step 11), the neurite endpoints are derived. Neurite-cell body attachment points are identified by first applying a mask generated by dilation of the binary cell body image (step 13) to identify the proximal neurite segments (step 14). The endpoints of these proximal neurite segments are determined by erosion (step 15) and subtraction (step 16). Endpoints within the original cell body mask are removed by subtraction (step 17) to yield the majority of neurite-cell body attachment points. Since this procedure depends on the presence of endpoints in the overlap region between the dilated cell bodies and neurite skeleton, it does not identify the rare events whereby two neurites emerge very close together from a single cell body, forming V-shaped attachments. Such attachments are morphologically similar to unattached neurites that contact neuronal cell bodies tangentially, and are interpreted by our analysis as no attachment, rather than two. Our analysis also does not track neurites that grow on top of neuronal cell bodies. Thus, unattached neurites that cross neuronal cell bodies are incorrectly interpreted as two attachment points.

The resulting binary images of neurites, neuronal cell bodies and neurite endpoints are quantified per field, and the following specific neuromorphological parameters are reported: total neurite length, total neuronal cell body area, average cell body cluster size, total number of cell bodies, number of neurite-cell body attachment points, and number of neurite endpoints. Quantification of the average signal intensity is also reported.

If neuronal cell body overlap is negligible (as is the case for low density primary hippocampal neurons) the reported neuronal cell body count should provide sufficient accuracy for most applications. However, if neuronal cell bodies form higher order clusters, as is commonly observed for neurons derived from P19 cells, the average neuronal cell body number can be estimated by dividing the total neuronal cell body area by a user-defined reference size of typical neuronal cell bodies. The ratio between the average cell body structure area and the measured area of individual cell bodies can serve as an approximate measure of neuronal cell body clustering.

The primary measurements generated by the NeuriteQuant tool are used to derive additional neuromorphological features, including total branch number, branch density along the neurite length, average neurite length per neuron and average length of individual neurites. For this purpose, numbers of branch points are deduced as the difference between neurite endpoints and neurite attachment points. This is valid as long as neurite and neuronal cell body detection is robust, as interrupted neurite segments or neurites that are separated from neuronal cell bodies also give rise to an increased difference between neurite endpoints and neurite attachment points. All measurements can be set up for multiple colour channels, facilitating parallel analysis of, for example, neuronal subtype morphologies or individual measurements for axons versus dendrites.

An increase in the ratio between total neurite length and neuronal cell body area can result from either increases in neurite outgrowth (increased total neurite length with constant neuronal cell body area) or from shrinkage of neuronal cell bodies (decreases in neuronal cell body area with constant neurite length), or both in combination. In our experiments, changes in the ratio between total neurite length and neuronal cell body area usually resulted from altered neurite outgrowth. Interestingly, taxol application to primary hippocampal neurons resulted in an increase in total neurite length and an apparent decrease in neuronal cell body area (see [7]). The apparent decrease in neuronal cell body area seemed to originate from increased microtubule bundling, resulting in a smaller cell body area as detected by antibodies against neuronal tubulin. Thus, careful review of experimental data by a human observer is essential to detect and interpret unexpected changes in neuronal morphology and their effect on automated analysis. The efficient web-browser based data review feature facilitates such *post hoc *data analyses.

## Results

### Measurement of Neurite Outgrowth in high density cultures of differentiating P19 stem cells

We first tested whether the neuronal morphology measurement algorithm in NeuriteQuant can extract neuromorphological features from differentiating mouse P19 cells, a valuable pluripotent, stem cell-like model for neuronal differentiation and neuritogenesis [8]. These cultures are usually grown at very high cell densities and they display a high degree of neurite overlap. We cultured P19 cells in plastic bottom, 384-well plates and induced their differentiation by transfection with the neurogenic transcription factor NeuroD2 [9]. In these conditions P19 cells form a dense population of neuronal cells, which extend neurites on top of a monolayer of non-neuronal cells. Cells were fixed 4 days after plating, stained for neuron-specific βIII-tubulin (using antibody TuJ1) and were imaged using an epifluorescence microscope.

Multicolour labelling could obviously facilitate morphological analysis of neurite length and neuronal cell body area (e.g. by using HuC/HuD as a marker for neuronal cell bodies [10]). However, our goal was to extract the maximal information from images captured using a single fluorophore. With this strategy, we retain maximum flexibility to multiplex markers of additional biological interest as shown in the next section on selective measurements on axons vs. dendrites. Therefore, we restricted our analysis in these initial experiments to a single fluorescence channel (using secondary Alexa 568-labeled antibodies to detect neuronal tubulin with antibody TuJ1).

In order to compare objective automatic analysis to subjective, manual tracing of neurites, we first measured neurite length both with NeuriteQuant and with the semi-automated tool NeuronJ [2]. The NeuronJ protocol requires that users can unambiguously assign neurite structures, which are then traced in an interactive fashion. Due to this interactive component, such measurements are subject to user bias - especially if weakly stained neurites are analyzed, which cannot be unambiguously distinguished from background signals.

**Figure 3:**
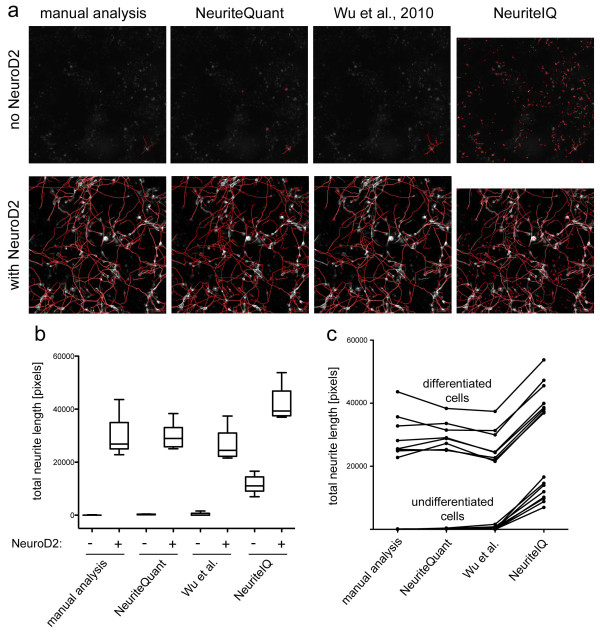
**Comparison of automated analysis with NeuriteQuant to manual analysis and existing software**. a) P19 cells were cultured with or without transfection of the neurogenic transcription factor NeuroD2. Neurite length was measured either by manual tracing using the semi-automated NeuronJ program [2], the NeuriteQuant package, the method by Wu et al. [11] or the NeuriteIQ software. b) Box and Whisker graphs from quantitative measurements of neurite length from 8 microscopic fields per condition. c) Corresponding automated and manual analyses of neurite length of identical images are symbolized by a connecting line.

As shown in Figure 3a, automatic and manual neurite length measurements were very similar. Quantitative analysis (Figure 3b) shows that measurement for neurite outgrowth was consistently low for undifferentiated cells (no NeuroD2) and consistently high for differentiated cells (with NeuroD2), both via manual and NeuriteQuant based analysis. Furthermore, both analyses were highly correlated as shown by paired measurement values in Figure 3c and quantitative analysis (Pearsons's r: 0.990). Importantly, automated analysis is rapid (<10 seconds per field of view using a low-end PC) and thus economically scalable to genome-wide assays.

### Comparison to existing Software

We next compared the performance of NeuriteQuant with other related analysis toolkits. A recently developed software tool [11] by Wu et al follows a similar strategy as our approach, by focusing on extracting neurite and neuronal cell body structures on a per frame basis. However, that tool does not provide analysis of neurite branching. The published approach, which is tailored towards analysis of neuronal cultures from Drosophila, is available from the authors upon request. The analysis algorithm of Wu et al is optimized to detect neurites with high accuracy, but it also requires more computational power (approximately four-fold slower than NeuriteQuant), which might be disadvantageous for large-scale analysis, such as in high-content screening campaigns. For quantitative comparisons, a modified neuronal soma detection method was applied (Pengyu Hong, personal communication). Although individual neurites that were not attached to the detected neuronal cell bodies were missed, neurite tracings of P19 cells obtained by the Wu et al. [11] method were highly accurate (Figure 3a) and quantitative measurements were similar to manual analysis (Figure 3b). Overall correlation with manual analysis was slightly improved compared to the faster NeuriteQuant method (Pearsons's r: 0.998).

Pool et al [12] developed a software tool called NeuriteTracer that is freely available. In contrast to NeuriteQuant, NeuriteTracer requires images of separated nuclei for quantification of average neurite length, and is thus less reliable at high densities of non-neuronal cells, such as in cultures of differentiating P19 cells. Huang et al [13] developed a related tool, called NeuriteIQ. This tool has similar features to NeuriteTracer, but is reported to be more accurate [13]. As shown in Figure 3a, the majority of neurites of P19 cells was detected by NeuriteIQ, however, using the settings available for the publicly available software package, false positive neurite segments were also frequently encountered, leading to consistently higher neurite length measurements even for undifferentiated cells (Figure 3b). Overall correlation between manual analysis and NeuriteIQ-based analysis was acceptable (see Figure 3c, Pearsons's r: 0.987), however, computation speed was much slower compared to NeuriteQuant (3 min per image for NeuriteIQ, vs. 10 sec/image for NeuriteQuant).

The Z-factor [14] is a measure for the dynamic range of quantitative measurements and therefore often used to evaluate assay quality. A Z-factor higher than 0.5 is characteristic of a robust assay, an assay with Z-factor below 0 is considered poor or unusable, and an intermediate value corresponds to a marginally useful assay. NeuriteQuant-based measurements of both undifferentiated and differentiated cells have a low standard deviation and the difference between the respective means is large. This high dynamic range is reflected in a high Z-factor of 0.53. The Z-factor of the measurements via the method by Wu et al. or obtained via manual analysis was slightly lower at 0.29, due to the smaller difference in means and larger standard deviations. In the case of NeuriteIQ-based measurements, the negative controls have a very high standard deviation and, therefore, the corresponding Z-factor is fairly low, at 0.087.

Taken together, NeuriteQuant trades off accuracy for speed as compared to the method of Wu et al. [11]. In comparison to NeuriteIQ, NeuriteQuant is both faster and more accurate. Importantly, in contrast to NeuriteIQ and the method by Wu et al [11], which are based on the commercial software package MATLAB, NeuriteQuant is based on the free software tool ImageJ. In addition, NeuriteQuant offers basic analysis of neurite branching, which is neither included in the method of Wu et al., nor part of the publically available version of NeuriteIQ. Finally, as compared to other free solutions, NeuriteQuant is unique due to the automatically generated, web-browser data review feature.

In comparison to freely available tools, commercial software solutions, such as HCA-Vision [15] can offer more detailed analysis on the basis of single cell measurements, and report detailed branch patterns in individual neurons. Such analysis can be beneficial for studying low-density cultures, in which neurite arbors are well separated between individual neurons. However, in dense neuronal cell populations, separation of individual cells is often not possible even by a trained, careful, human observer. In such situations, the association of measurements with individual cells is often arbitrary and might even be misleading. A summary of the quantitative measurements we performed on high-density differentiated P19 cells is given in Table 1.

**Table 1:** Summary of comparison to manual analysis and existing software

	manual analysis	NeuriteQuant	Wu et al.	NeuriteIQ
correlation to manual analysis (Pearson's r)	N/A	0.990	0.998	0.987
Z-factor	0.29	0.53	0.29	0.087
analysis time (one 1k × 1k image)	>1 h	10 sec^1 ^8 sec^2^	40 sec^2^	3 min^1^
software requirements	ImageJ (free) NeuronJ (free)	ImageJ (free)	MATLAB (commercial)	MATLAB (commercial)
availability	free	free, open-source	upon request to author	compiled program available online

^1^: 32-bit WindowsXP on low-level Pentium notebook. ^2^: 64-bit Windows 7 on intermediate-level quad-core desktop.

Analysis speed is of particular interest, given the increasing availability of genome-wide libraries that enable functional assays amenable to high throughput automation. Therefore, as a proof of principle, we applied our automated assay in P19 cells to a focused pilot screen using RNA interference mediated gene knock-down [16]. In this focused screen, NeuriteQuant was able to identify siRNA oligonucleotides that in a dose dependent manner either a) increased neurite outgrowth (as seen with knockdown of WASP family protein Wasf1), b) decreased neurite outgrowth (as seen with knockdown of dynein subunit Dctn1) or c) decreased neuronal differentiation (as seen with knockdown of the small GTPase RhoA) (data not shown). These results are in agreement with earlier studies in which these isoforms or related genes were inhibited [17-23], and thus validate NeuriteQuant's application for genomic screens.

### Measurement of Axon and Dendrite Outgrowth and Branching from Primary Hippocampal Neurons

Next we tested whether our analysis protocol is applicable to quantification of more complex morphology of primary neurons. Hippocampal neurons are a well-established model system for studying neuronal development and function [24]. They form two functionally distinct neurite types, axons and dendrites, both of which display complex, branching arbors. We grew mixed neuron/glia cultures from rat hippocampus in plastic bottom 384-well plates and applied a series of drugs to examine how primary neurons are affected by disruption of cytoskeletal components. We also applied the transcriptional inhibitor actinomycin D to determine how assay measurements are affected by a generally toxic compound that potentially generates cell debris and dystrophic structure. To extract additional information regarding axon and dendrite specification, we double-stained neurons with antibody TuJ1, which labels both axons and dendrites, and antibody to MAP2, which specifically labels only dendrites [25].

**Figure 4:**
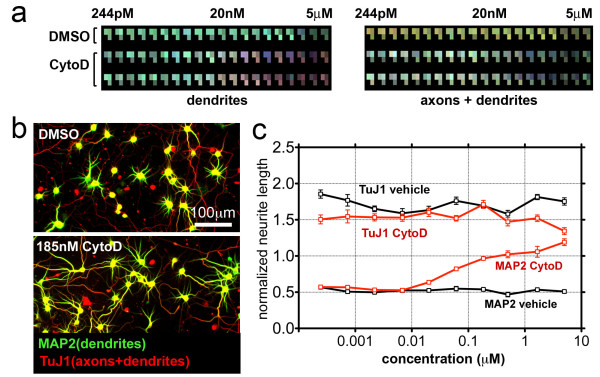
**Measurement of axonal and dendritic outgrowth from primary neurons**. Dissociated hippocampal neurons were cultured in 384-well plates and incubated with the indicated compounds for 3 days, starting one day after plating. a) Heatmaps were automatically generated by NeuriteQuant to summarize the main measurements: neurite length (red), neuronal cell body area (green) and mean neuronal marker signal intensity (blue). The left heatmap shows these measurements for dendrites only, detected using MAP2, and the right heatmap shows the corresponding measurements for total neurites (axons plus dendrites) using the general neurite marker TuJ1. The shift in colour hue from green to red with increasing cytochalasin D concentrations in the left heatmap indicates a dose-dependent shift in the ratio between neurite length and neuronal cell body area. b) Representative automatically acquired images of neurons incubated with either vehicle (DMSO) or cytochalasin D prior to staining with TuJ1 (axons plus dendrites; red) and MAP2 (dendrites only, green); yellow indicates regions of overlap in the merged image. c) Quantitative analysis of cytochalasin D dose response curves demonstrates a >50% increase in the normalized neurite length (the ratio of total neurite length to total neuronal cell body area per field), as based on the dendritic marker MAP2. In contrast, analysis based on the general neurite marker TuJ1 shows a slightly opposite trend, because axon length decreased in response to cytochalasin D (not shown). Corresponding amounts of vehicle had no significant effect on neuronal morphology as detected using either marker. Data represent mean ± standard error of 9 image fields from three independent repetitions per condition.

Figure 4a shows an integrative graphical representation of multiple measurements from this small-scale compound screen in the form of a heatmap. Such heatmaps are displayed in the NeuriteQuant data-browser and offer an easily accessible mode to visualize, compare, and display experimental results. The individual colour components in our heatmaps represent the magnitude of corresponding signal measurements and are displayed as normalized shades of gray: Black represents no signal, grey (colour value 128) represents the average measurement value of the given plate and white (colour value 255) represents 2x average of the given plate. The shades of grey from three quantitative measurements are then combined as colour components red, green, and blue into each heatmap cell. In this example, each of the two heatmaps display measurements of total neurite length in red, total neuronal cell body area in green and the mean marker intensity in blue - either measured via the dendrite marker MAP2 or via the total neurite marker TuJ1.

A dose-dependent stimulatory effect of cytochalasin D on dendrite outgrowth, which is consistent with an earlier report [26] is clearly visualized in the heatmap for dendrite measurements as a change in the colour component ratio from green towards red (Figure 4a). This change in colour component ratio is not seen in the heatmap for measurements of axons and dendrites. Figure 4c shows that extensive dose response curves with small error bars can be generated from these measurements - a feature that is essential for HCS, and that would be laborious with manual methods.

**Figure 5:**
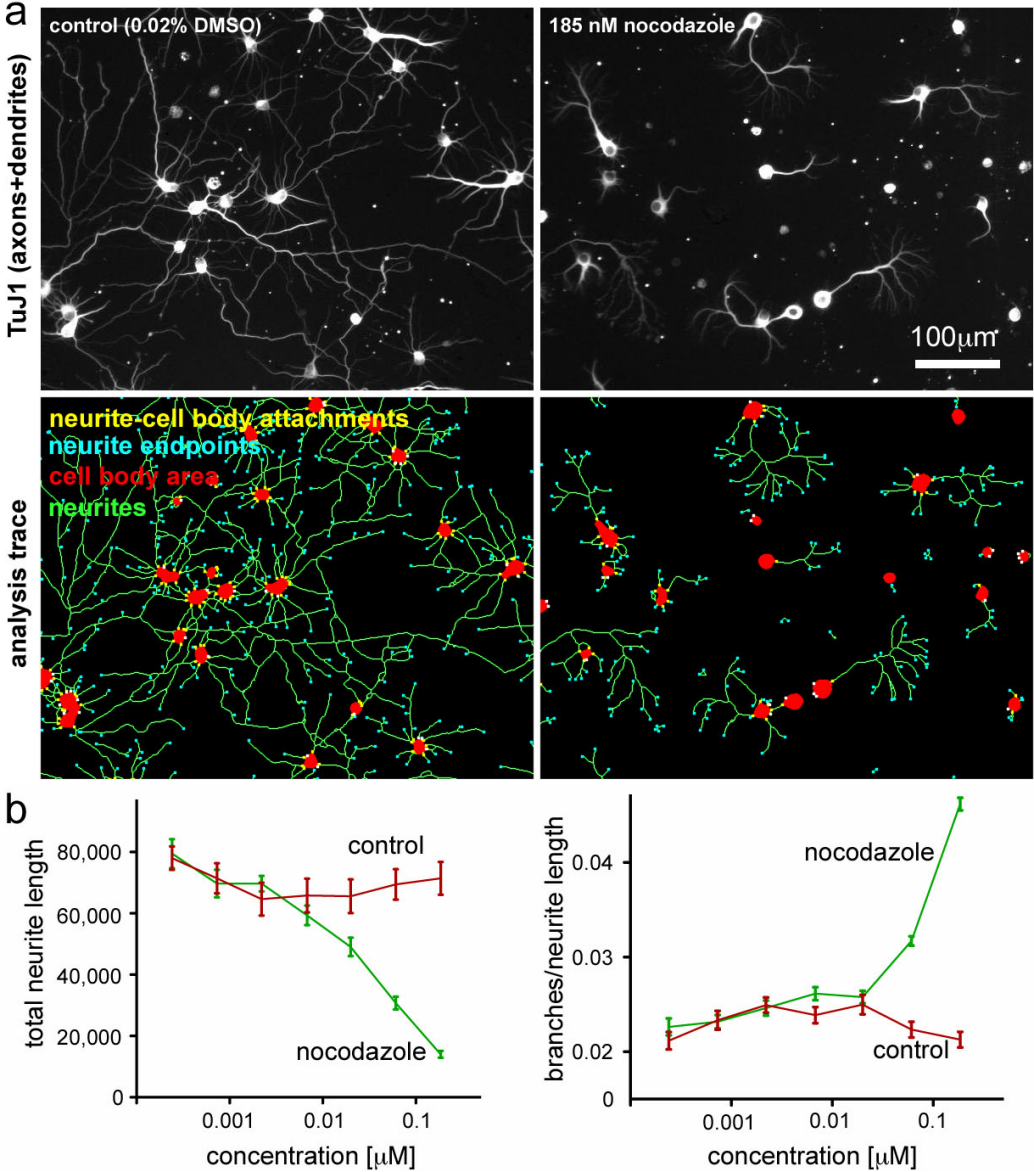
**Measurement of axonal and dendritic branch density from primary hippocampal neurons**. Neurons were treated with vehicle (DMSO), or nocodazole for three days starting one day after plating. Neurons were stained with the neuronal marker TuJ1. To quantify the average density of neurite branches, the following formula was used: (neurite cell body attachment points - neurite endpoints)/neurite length. a) Representative images and analysis traces of control and treated neurons show decreased neurite length and increased branch density in the presence of nocodazole. b) Quantification of nocodazole titration shows a dose-dependent decrease in total neurite length but an increase in branch density of TuJ1-positive neurites. In our experimental regime, we detected increases in neurite branching at concentrations between 61-185 nM. Measurements from higher concentrations were not included in our analysis, as cell viability and neurite outgrowth decreased drastically, and measurement artefacts from cell debris prevented reliable determination of branch density.

In agreement with previous studies [27,28], we also detected a dose dependent decrease in branch density with taxol (not shown) and an increase in neurite branch density with intermediate concentrations (61-185 nM) of nocodazole (Figure 5). Manual counting of branch points using the cell counter tool of ImageJ and NeuriteQuant analysis both detect an approximately two-fold increase in branch density after treatment with 185 nM nocodazole (manual counting, control = 0.0094 ± 0.0009 branches/pixel; manual counting, 185 nM nocodazole = 0.0202 ± 0.0033 branches/pixel; NeuriteQuant, control = 0.0200 ± 0.0019 branches/pixel; NeuriteQuant, 185 nM nocodazole = 0.0351 ± 0.0045 branches/pixel; n = 3 images per condition; only branches were counted and neurite crossings were ignored in manual analysis). Although NeuriteQuant analysis consistently detects a higher total number of branches, automated measurements were reproducible as seen by the small error bars (Figure 5b) and therefore NeuriteQuant is able to detect overall changes in branch density on a per field basis. Correlation between manual counting and automated analysis was weaker (Pearsons's r: 0.8405) compared to the simpler neurite length analysis, but it was nevertheless statistically significant (p = 0.036). The higher number of branches detected by NeuriteQuant is mostly due to interrupted neurite segments, which give rise to false positive branch detection, as well as dim branches that are easily missed in manual analysis. At high concentrations of nocodazole or actinomycin D, quantification of branch density was not accurate, as overall cell viability and neurite outgrowth declined drastically, giving rise to many detected neurite fragments, which were disconnected from detected cell bodies. The complete, automatically generated browser for this dataset, which can be navigated via several interactive heatmap variants and interactive 2D-plots, is available on the NeuriteQuant website [5].

**Figure 6:**
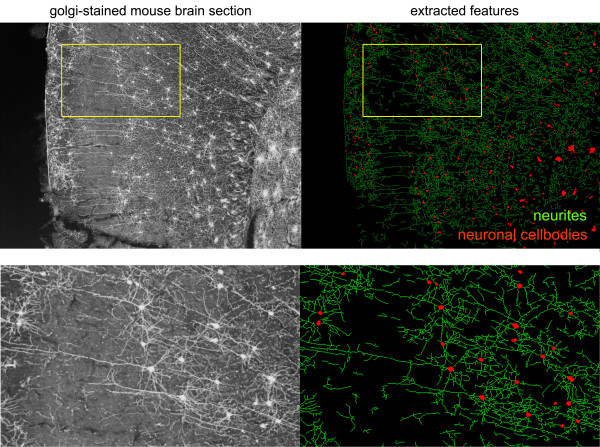
**Analysis of neuronal morphology in Golgi-stained mouse brain sections**. Wild-type P26 mouse brains were stained using modified Golgi-Cox impregnation (FD NeuroTechnologies). NeuriteQuant is able to extract most features in images of silver-stained neurons that display a clearly defined soma and dendritic arbor (see enlarged region). During sectioning, neurites are often separated from their parent cell, therefore branching was not evaluated.

Finally, we tested whether NeuriteQuant was compatible with analysis of neurons in brain sections. Figure 6 shows automated analysis of an inverted image of a Golgi-stained section from mouse cortex. Most neuronal cell bodies and neurites were reliably detected, despite the relatively large variation in background staining within these sections.

### Limitations of NeuriteQuant

In the design of NeuriteQuant, our aim was not to build a tool that corrects all potential problems that might occur during data acquisition. We rather aimed for a simple, straightforward implementation that offers robust and fast analyses. Due to this simplicity, NeuriteQuant has clearly defined limitations that need to be kept in mind concerning the interpretation of measurements:

1) Image analysis is optimal at high signal-to-noise levels, which enable detection of weak neurite structures by setting low detection thresholds. Therefore, markers that are highly expressed in neurons, such as the neuronal βIII tubulin isoform (antibody TuJ1) or the dendritic marker MAP2 are preferable. Measurements via markers that stain neurites barely above noise levels are less accurate and in such suboptimal conditions, weaker neurite structures might be excluded from analysis by the thresholding procedure.

2) At high cell densities, if neuronal cell bodies are not separated from each other, the neuronal cell body number can only be estimated by dividing the total neuronal cell body area by a user-defined reference size of typical neuronal cell bodies. In extreme cases (for example, if unusually large cell aggregates are encountered) this estimation may become inaccurate. NeuriteQuant also cannot distinguish individual neurites within fasciculated bundles and reports only on the length of apparent neurite structures, whether they are made of a single or multiple, bundled neurites.

3) More complex morphometric measurements, which are derived from and/or dependent on the ratios of multiple primary morphological measurements, such as the average branch density, are less accurate if only few cells or few, small neurite fragments are analyzed per field. For example, if cell viability and neurite outgrowth is drastically reduced, any falsely identified neurite segment that is disconnected from a neuronal cell body gives rise to false positive detection of neuronal branches and can therefore strongly influence the measurement of neuronal branch density, as seen in primary hippocampal neurons treated with high doses (>185 nM) of nocodazole. It is therefore imperative that such complex measurements are always interpreted in the context of successful and reliable primary measurements, and verified carefully by the user via the web-based data browser.

It should be noted that many conditions that are not readily quantified by the existing NeuriteQuant software can nonetheless be identified on the basis of primary measurements. Uninterpretable images could then either be excluded, or analysis settings could be refined to extract biologically useful information. For example, large cell aggregates can be detected by measuring the average cell size. By setting an appropriate threshold, such potentially misleading images could be identified. Subsequently they could be removed if deemed an artefact, or, if they are of interest to the user, NeuriteQuant based processing could be used to capture such features for quantitative analysis.

### Ongoing Development

NeuriteQuant is implemented as an ImageJ macro, and therefore easily accessible for extension. Updates to the NeuriteQuant tool will be made available on the NeuriteQuant website [3]. The current version already supports import of large, complex datasets produced by automated screening microscopes. For example, import routines to directly access images produced by the ScanR system (Olympus, Hamburg) or custom journals implemented in the microscopy control software Metamorph (Molecular Devices, Inc.) are implemented in the current version of NeuriteQuant. Due to its open-source implementation, the NeuriteQuant image analysis pipeline can be adapted to any well-defined naming convention and it can use any input format that is supported by ImageJ. Similarly, further image pre-processing can be performed, or additional morphological features can be analysed by combining the streamlined image and data management aspects of the NeuriteQuant toolset with the varied and extendable capabilities of ImageJ. Thereby, NeuriteQuant not only represents a framework for the specialized morphometric analysis of neuronal development as shown here, but also provides a valuable starting point for development of other morphometric analyses. Detailed instructions for the modification and extension of NeuriteQuant are given in the NeuriteQuant script file.

## Conclusions

In conclusion, we offer NeuriteQuant as a free, open-source toolkit for rapid analysis of neuronal morphology. NeuriteQuant measurements provide a meaningful characterization of neuronal morphology and they can be used to identify a wide range of morphological changes with high sensitivity. By explicitly focusing on a "per-field" analysis strategy, our method avoids ambiguities in defining single neurons and their respective neuronal arbors within a dense population of neurons, which is a typical situation for many neuronal culture systems. NeuriteQuant is especially effective in deducing neuronal parameters from relatively low-resolution images (10x) and is thus able to rapidly quantify neuronal morphology from large neuronal populations. We anticipate that NeuriteQuant will facilitate the discovery of new pathways and molecular targets in neuronal development and regeneration.

## Availability and requirements

### Project name

NeuriteQuant

### Project home page

http://www.chemie.uni-dortmund.de/groups/CB/bastiaens/dehmelt/NeuriteQuant/

### Operating system(s)

Windows/PC, MacOS

### Programming language

Java/ImageJ script

### Other requirements

ImageJ 1.38 or higher (see documentation for details)

### License

GNU GPL

### Any restrictions to use by non-academics

GNU GPL (no additional restrictions)

## Authors' contributions

SH supervised the study. LD and SH designed and interpreted experiments and wrote the manuscript. LD conceived the analysis strategy; LD, GP, and EH performed experiments and analyzed data. All authors have read and approved the final manuscript.

## Supplementary Material

Additional file 1 **Text file containing a summary of the image processing algorithm and Experimental Procedures**. Click here for file
