# Supplementary material for: NeuriteQuant: An open source toolkit for high content screens of neuronal Morphogenesis
Source: BMC Neurosci. 2011 Oct 11;12:100. doi: 10.1186/1471-2202-12-100 (PMC3208608; doi:10.1186/1471-2202-12-100)
Supplement: Additional file 1 — Text file containing a summary of the image processing algorithm and Experimental Procedures. [file 1471-2202-12-100-S1.DOC]

**Summary of Image Processing Algorithm:**

*Parameters:*

1.approximate neurite diameter for morphological filtering: d

2. intensity threshold for detection of neuronal cell bodies: T_CB_

3. intensity threshold for detection of neurites: T_N_

4. size limit for particle removal: s

*Procedure 1: Extraction of cell bodies*

1. load "raw image"

2. perform morphological open filter with circular structuring element (size d ~ neurite diameter)

3. binarize image with predefined intensity threshold T_CB_

Result: "cell bodies"

*Procedure 2: Extraction of neurites*

1. load "raw image"

2. perform morphological open filter with circular structuring element (size d ~ neurite diameter)

3. subtract filtered image from "raw image"

4. binarize image with predefined intensity threshold T_N_

5. remove particles smaller than preset limit s

6. skeletonize image - the resulting image is called "skeleton"

7. identify overlap with image "cell bodies" (from Procedure 1) by pixel-wise logical AND operation

8. subtract overlap from image "skeleton"

Result: "neurites"

*Procedure 3: Extraction of endpoints*

1. load image "skeleton" (from Procedure 2)

2. remove terminal pixels by binary erode function

3. subtract eroded image from the image "skeleton" - the resulting image is called "neurite segment termini"

4. identify overlap with image "cell bodies" (from Procedure 1) by pixel-wise logical AND operation

5. subtract overlap from image "neurite segment termini"

Result: "endpoints"

*Procedure 4: Extraction of attachment points*

1. load image "cell bodies" from Procedure 1

2. enlarge structures by binary dilate function

3. identify overlap with image "skeleton" (from Procedure 2) by pixel-wise logical AND operation - the resulting image is called "proximal neurite segments"

4. remove terminal pixels by binary erode function

5. subtract eroded image from the image "proximal neurite segments" - the resulting image is called "proximal neurite segment termini"

6. subtract the image "cell bodies" (from Procedure 1) from the image "proximal neurite segment termini"

Result: "attachment points"

**Experimental Procedures:**

### *Cell Culture and Transfection*

Plasmid pIRES2-EGFP-NeuroD2 was generated by subcloning of NeuroD2 from EcoRI/SnaBI cut pCS2+NeuroD2 (gift from David Turner, University of Michigan) into EcoRI/SmaI cut pIRES2-EGFP (Clontech, Mountain View, CA).

P19 cells were obtained from ATCC (Teddington, UK) and cultured in growth medium (clear MEM+glutamate/pyruvate, 10% FBS and penicillin/streptomycin; all media components were obtained from Invitrogen, Carlsbad, CA). For experimental manipulations, P19 cells were grown in flat bottom 384-well plates (Art.-Nr.: 781092; Greiner, Monroe, NC). For differentiation in the absence of siRNAs, P19 cells were reverse transfected with NeuroD2 using Fugene-6 (Roche, Indianapolis, IN). Briefly, per well of a 384-well plate, 40 ng pIRES2-EGFP-NeuroD2 were added in 7.5 μL clear MEM. Next, 150 nl Fugene-6 was added in 7.5 μL clear MEM. Plates were incubated for 20-30 minutes and 4000 P19 cells were plated per well in 85 μL serum reduced medium (growth medium with 5% FBS without penicillin/streptomycin).

For cotransfection with siRNA oligonucleotides, a modified procedure using Lipofectamine 2000 (Invitrogen, Carlsbad, CA) was used. For this procedure, the indicated amounts of Flexiplate siRNA oligos or controls (Qiagen, Valencia, CA) were spotted onto 384 well plates in 5 μL clear MEM. Spotted plates were kept at –80°C and thawed immediately before use. 120 ng pIRES2-EGFP-NeuroD2 was added in 5 μL clear MEM followed by 120 nl Lipofectamine 2000 in 5 μL clear MEM (preincubated for 5 minutes according to manufacturer’s instructions). As in the Fugene-6 based protocol, plates were incubated for 20-30 minutes and 8000 P19 cells were plated per well in 85 μL serum-reduced medium. Taxol was obtained from (Calbiochem, CA). Unless stated otherwise, fine chemicals and pharmacological inhibitors were obtained from Sigma, St. Louis, MO.

Primary rat hippocampal neurons were prepared as described^1^. Neurons were plated at 4800 cells/well on flat bottom 384-well plates, which were pretreated by overnight application of poly-L-lysine (100 μg/mL).

#### Immunohistochemistry

P19 cells or neurons were grown for 4 days and fixed using 4% formaldehyde at 37°C for 20 min. Cells were permeabilized using 0.25% Triton-X-100 for 15 minutes, followed by blocking in 10% BSA and primary and secondary antibody incubation in 2% BSA. Mouse monoclonal anti-beta tubulin-III antibodies (Tuj1, a gift from Anthony Frankfurter, University of Virginia) were used at 1:2000 dilution. Rabbit, anti-MAP2 antibodies (antiserum 266) were used at 1:2000. Alexa 568-labled anti-mouse antibodies and Alexa 488-labeled anti-rabbit antibodies (both from Invitrogen, Carlsbad, CA) were used as secondaries at 1:1000 dilution. For autofocusing, cells were counterstained with DAPI (Sigma, St. Louis, MO).

#### Automated Microscopy

Automated imaging was performed on an Axiovert 200M microscope (Zeiss, Thornwood, NY) equipped with a 10x Fluar Objective (0.50 NA) and a motorized stage (Ludl, Hawthorn, NY). Automated scanning of 384-well plates was done using a custom journal written for Metamorph (Universal Imaging Corporation, West Chester, PA). The custom journal is available upon request. Briefly, the DAPI channel was used to find the focal plane containing cell bodies. Subsequently, cells were imaged using the red and green fluorescent channels. To ensure optimal spectral separation, channels were selected using an automated reflector turret equipped with sets of excitation/emission filters and dichroic mirrors (Chroma) optimized for each individual colour. Images were automatically saved and named based on their position of the 384-well plate in a format compatible with automated analysis.

**Supplementary References:**

1. Y. Shiraishi, A. Mizutani, S. Yuasa et al.*, J Neuroch*e**m** 87 (2), 364 (2003).
